# Integrating generative AI with ABCDE rule analysis for enhanced skin cancer diagnosis, dermatologist training and patient education

**DOI:** 10.3389/fmed.2024.1445318

**Published:** 2024-10-03

**Authors:** Lennart Jütte, Sandra González-Villà, Josep Quintana, Martin Steven, Rafael Garcia, Bernhard Roth

**Affiliations:** ^1^Hannover Centre for Optical Technologies, Leibniz University Hannover, Hannover, Germany; ^2^Coronis Computing S.L., Girona, Spain; ^3^Institute of Computer Vision and Robotics Research, Universitat de Girona, Girona, Spain; ^4^Cluster of Excellence PhoenixD, Leibniz University Hannover, Hannover, Germany

**Keywords:** melanoma, ABCDE rule, artificial intelligence, patient education, sequential dermoscopy

## Abstract

**Significance:**

The early detection and accurate monitoring of suspicious skin lesions are critical for effective dermatological diagnosis and treatment, particularly for reliable identification of the progression of nevi to melanoma. The traditional diagnostic framework, the ABCDE rule, provides a foundation for evaluating lesion characteristics by visual examination using dermoscopes. Simulations of skin lesion progression could improve the understanding of melanoma growth patterns.

**Aim:**

This study aims to enhance lesion analysis and understanding of lesion progression by providing a simulated potential progression of nevi into melanomas.

**Approach:**

The study generates a dataset of simulated lesion progressions, from nevi to simulated melanoma, based on a Cycle-Consistent Adversarial Network (Cycle-GAN) and frame interpolation. We apply an optical flow analysis to the generated dermoscopic image sequences, enabling the quantification of lesion transformation. In parallel, we evaluate changes in ABCDE rule metrics as example to assess the simulated evolution.

**Results:**

We present the first simulation of nevi progressing into simulated melanoma counterparts, consisting of 152 detailed steps. The ABCDE rule metrics correlate with the simulation in a natural manner. For the seven samples studied, the asymmetry metric increased by an average of 19%, the border gradient metric increased by an average of 63%, the convexity metric decreased by an average of 3%, the diameter increased by an average of 2%, and the color dispersion metric increased by an average of 45%. The diagnostic value of the ABCDE rule is enhanced through the addition of insights based on optical flow. The outward expansion of lesions, as captured by optical flow vectors, correlates strongly with the expected increase in diameter, confirming the simulation’s fidelity to known lesion growth patterns. The heatmap visualizations further illustrate the degree of change within lesions, offering an intuitive visual proxy for lesion evolution.

**Conclusion:**

The achieved simulations of potential lesion progressions could facilitate improved early detection and understanding of how lesions evolve. By combining the optical flow analysis with the established criteria of the ABCDE rule, this study presents a significant advancement in dermatoscopic diagnostics and patient education. Future research will focus on applying this integrated approach to real patient data, with the aim of enhancing the understanding of lesion progression and the personalization of dermatological care.

## 1 Introduction

Melanoma, a highly aggressive form of skin cancer, is responsible for the majority of skin cancer-related deaths worldwide (1). The incidence of melanoma has been steadily increasing over the past few decades, making it a significant public health concern. Early detection of melanoma is crucial, as it dramatically improves patient survival rates. Studies have shown that the 5-year survival rate for patients diagnosed with early-stage melanoma exceeds 90%, compared to less than 20% for those diagnosed at an advanced stage (2). Early diagnosis allows for less invasive treatments, reduces the need for extensive surgical procedures, and lowers healthcare costs associated with late-stage treatments (3). The integration of digital technologies, e.g., smartphone apps, represents an impactful advancement in training for melanoma diagnosis (4). The increasing significance of digital technologies in dermatology is highlighted by developments in contactless dermoscopy (5–8) and computerized analysis of pigmented skin lesions (9). Improvements in image quality enhance the visibility of dermoscopic patterns, providing more detailed information that can be instrumental in understanding lesion growth patterns and aiding in more accurate diagnoses. Other novel diagnostic modalities for melanoma are optical coherence tomography (10, 11), Raman spectroscopy (12–16), combined ultrasound and photoacoustic imaging (17, 18), and molecular diagnostics (19).

We propose the application of Cycle-Consistent Adversarial Networks (Cycle-GANs) in the transformation of dermoscopic images of nevi into simulated melanoma counterparts. This technology allows to visually demonstrate the subtle differences between nevi and melanoma using actual dermoscopic images from the patient’s body. During skin screening procedures, dermatologists could present both the original nevus image and the AI-generated melanoma version to the patient. This side-by-side comparison might aid dermatologists in explaining why certain lesions do not require excision and what changes to look for in follow-up examination (20–22). Conversely, for lesions appearing to be melanoma, the AI can generate a nevus counterpart to also help educate patients on recognizing the differences between nevi and melanomas and the importance of monitoring for changes. Additionally, the utilization of Cycle-GANs to simulate the evolution of skin lesions presents an innovative opportunity to test potential novel diagnostic criteria based on image processing against AI-generated simulations. By comparing the characteristics of nevi and simulated melanoma counterparts, our approach could accelerate the validation process of image processing techniques and might enhance the general understanding of lesion growth patterns and dynamics.

### 1.1 Application of GANs in dermatology

As artificial intelligence (AI) rapidly advances, its integration into dermatology has been mainly through Convolutional Neural Networks for skin cancer classification (23) and the implementation of explainable AI (24–26) in those classifications. Another network type of growing importance in dermatology are Cycle-Consistent Adversarial Networks (Cycle-GANs) (27). A GAN functions with two neural networks: a generator creating images and a discriminator evaluating them, producing increasingly realistic results. A Cycle-GAN links two GANs for bidirectional data transformation between domains. GANs have been increasingly employed in dermatology for various applications. GANs have already found a significant application in dermatology, particularly for data augmentation in skin cancer classification models (28). The classification models are limited by imbalances in the training datasets. The implementation of GANs for data augmentation to balance the training data enhanced the robustness and accuracy of these diagnostic models (29). GANs have also been used for color constancy in medical imaging, ensuring consistent appearance in dermatological images under different lighting conditions (30, 31). Color variability can lead to bias of dermatologists and impact the diagnosis. Generative models are effective in generalizing dermoscopic image appearance (31). Furthermore, Cycle-GANs have been utilized to transform dermoscopic images of melanoma into art works as a form of art therapy for melanoma patients (32). Despite these advancements, the simulation of disease progression, such as the transformation of nevi into melanoma, remains underexplored. Our study represents the first attempt to apply Cycle-GANs to simulate skin lesion progression, offering a novel approach to visualize potential changes in lesions over time.

## 2 Methods

**Figure 1:**
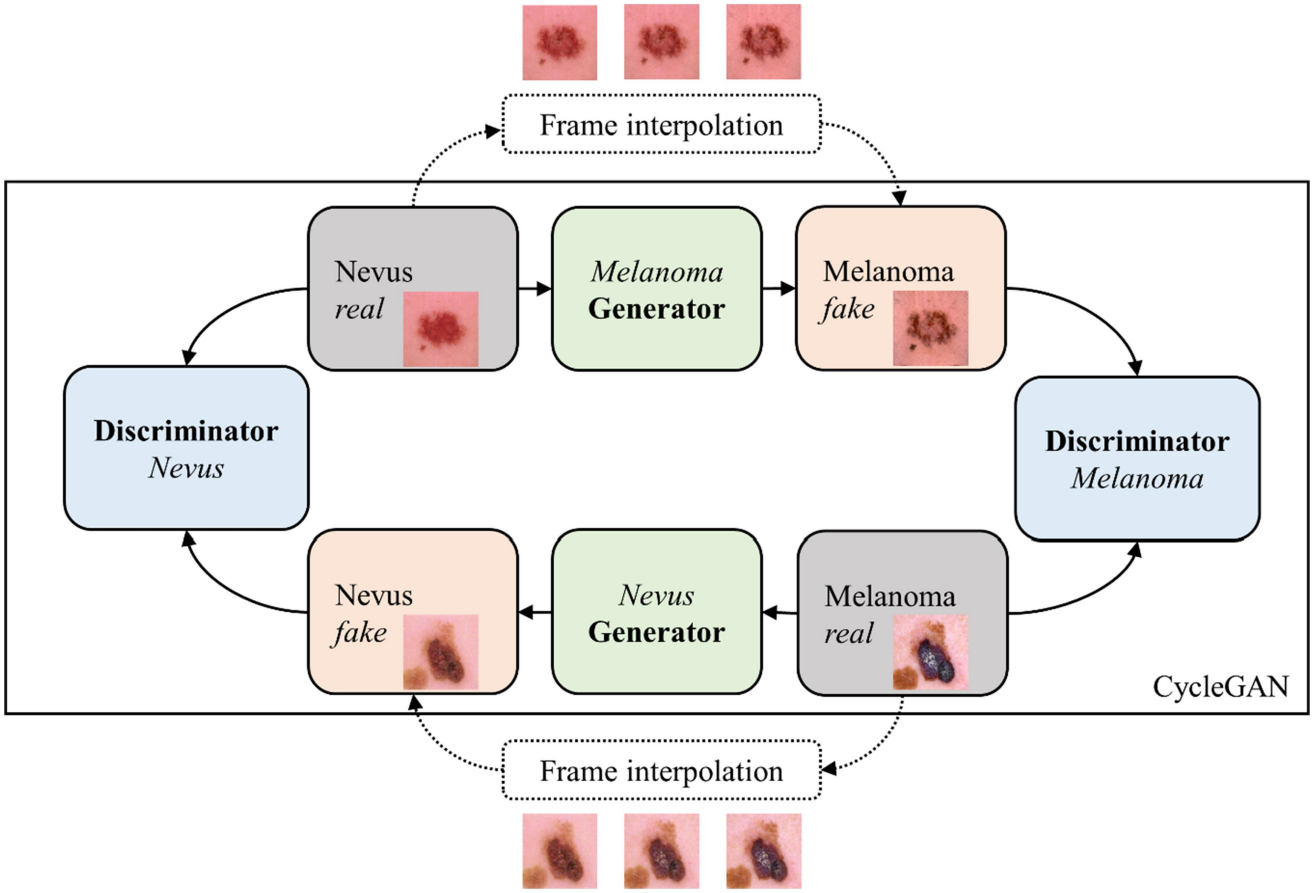
Cycle-GAN and frame interpolation scheme used to simulate lesion progression.

Cycle-GANs are well suited for the task of unpaired image-to-image translation. They enable the translation of images between two distinct domains without the necessity of one-to-one corresponding image pairs in the training data. This feature makes them particularly advantageous for dermatological applications, where there is often a significant imbalance between the number of nevus and melanoma lesion images available (33). The Cycle-GAN architecture consists of two generator networks and two discriminator networks. Each generator is tasked with translating images from one domain to the other. Each discriminator works to differentiate between actual and translated images within its respective domain. This dual setup enables the bidirectional translation capability of Cycle-GANs. Figure 1 shows a schematic of the Cycle-GAN and frame interpolation for the lesion progression simulation.

Grey panels indicate real images from a dermoscopic dataset, blue panels represent the discriminator model assessing the authenticity of images, green panels signify the generator model creating synthetic images, and orange panels highlight the generated synthetic images. This scheme describes the process of transforming nevi into their simulated melanoma counterparts through iterative learning and frame interpolation.

The model was trained using 1,571 melanoma images and 1,571 nevus images from the SIIM-ISIC dataset (34). A batch size of 1 was used, with 200 iterations and a learning rate starting at 0.0002, linearly decreasing to 0 after 100 iterations. This training condition is similar to the original Cycle-GAN publication (27). The images were resized to 286 × 286 pixels and randomly cropped to 256 × 256 before training to standardize input and reduce computational complexity. The generator is based on the ResNet architecture with 9 convolutional blocks, and the discriminator uses the PatchGAN architecture (27).

**Figure 2:**
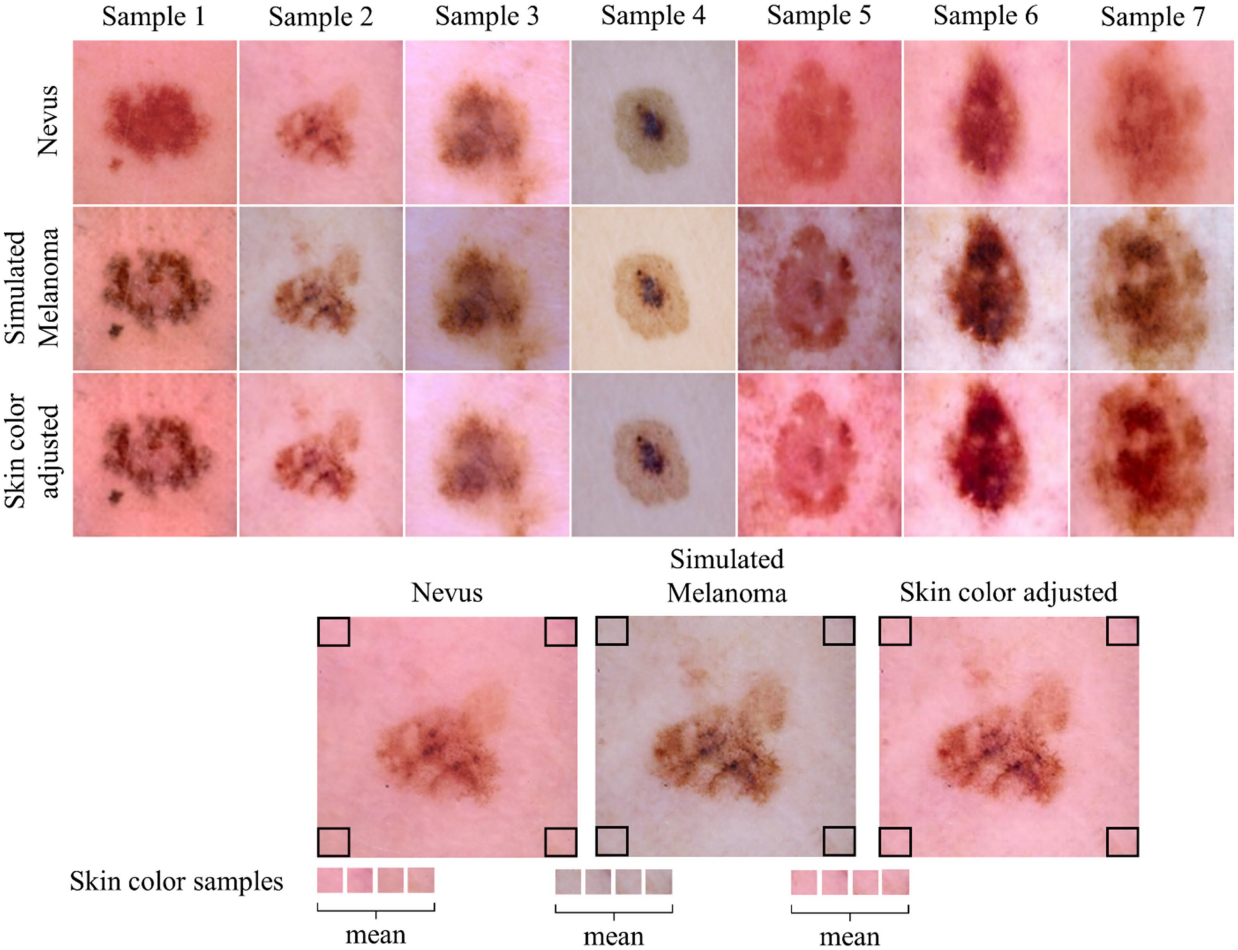
Overview of skin color adjustment. The first row displays the original nevus image, while the second row shows the initial output directly from the Cycle-GAN. The third row presents the color-adjusted output following post-processing. The lower part of the figure provides a visualization of the color adjustment process applied during post-processing.

Following the initial generation of simulation frames, a post-processing step is implemented to address color inconsistencies. The entire simulation is completed within seconds and can be efficiently performed on a standard PC. The original simulations produced by the Cycle-GAN and the frame interpolation alter the color of the surrounding healthy skin, complicating frame comparison concerning the color parameter of the ABCDE rule (35). To mitigate this issue and ensure comparability, the colors in each frame are normalized to match those of the first frame in the sequence using a straightforward color offset adjustment. This is achieved by extracting and averaging the RGB values from the corners of the real image in the simulation and then converting these values to the LAB color space to establish a baseline color reference (36). The corner samples only contain healthy skin. For each subsequent frame in the simulation, the deviation in average corner color from the reference is calculated. The color adjustment is applied to the entire image in the LAB space. Finally, the adjusted image is converted back to RGB for consistent visual analysis across the simulation. This process ensures that color changes in the lesion can be accurately assessed, while maintaining the consistency of the healthy skin from the first frame of the simulation to the last one, as displayed in Figure 2.

Utilizing frame interpolation, we create a seamless and gradual transition from the original dermoscopic nevus image to the simulated melanoma counterpart. This results in a brief video, illustrating the subtle progression of skin changes, potentially enhancing patient understanding of melanoma indicators. The frames demonstrate the transformation from the dermoscopic nevus image to an artificially simulated melanoma counterpart.

**Figure 3:**
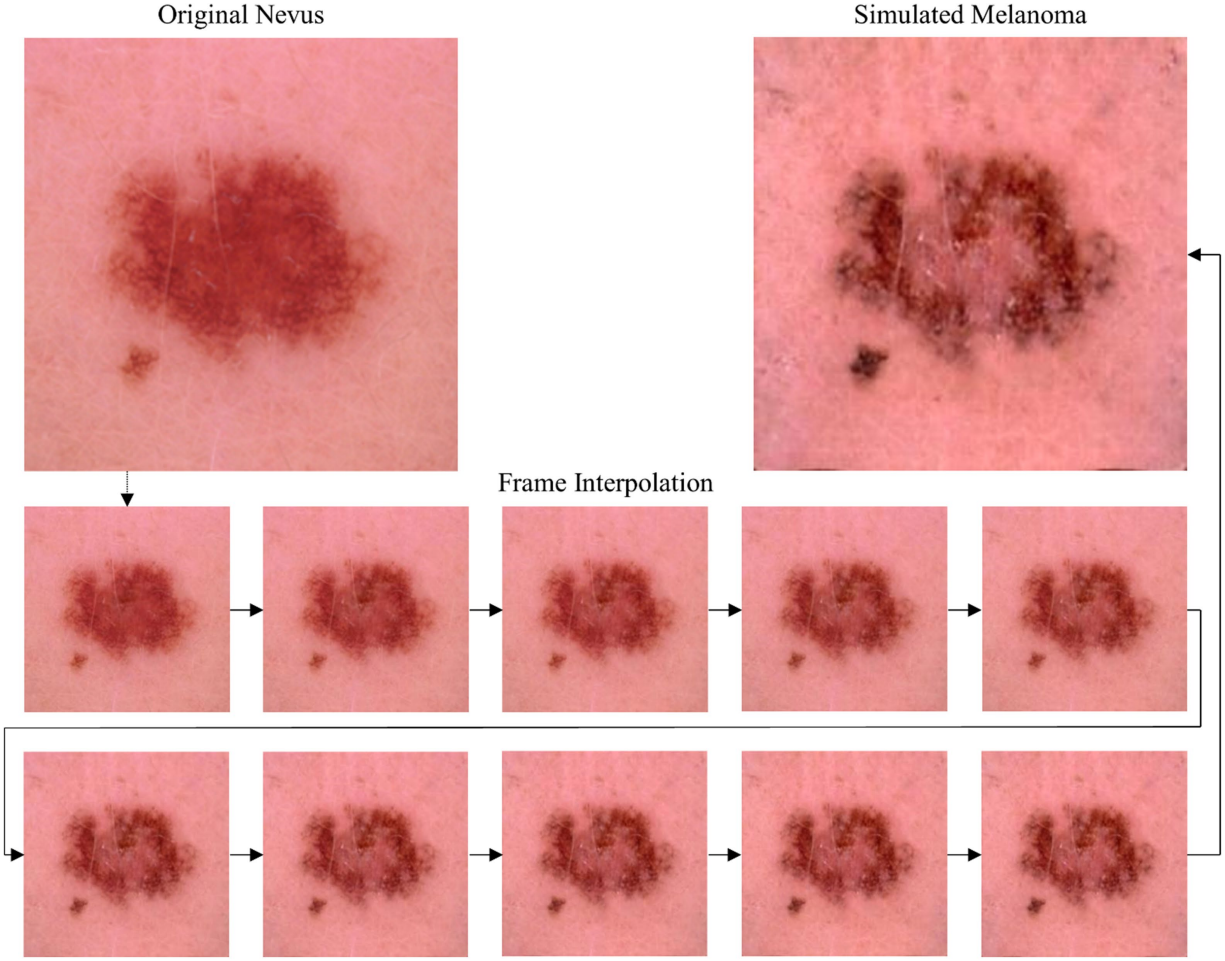
Top: Original nevus image from ISIC dataset (left) and AI-generated image (right) showing the lesion’s potential melanoma progression. Bottom: Selected frames from the frame interpolation video generated with Runway (2024 Runway AI, Inc., New York, United States) showing a stepwise progression from the nevus state to the simulated melanoma state.

Figure 3 shows a dermoscopic image of a nevus from the ISIC dataset (34) and a simulated melanoma counterpart as well as selected frames from a frame interpolation, showing the gradual transformation from the original nevus image to the simulated melanoma lesion image.

**Figure 4:**
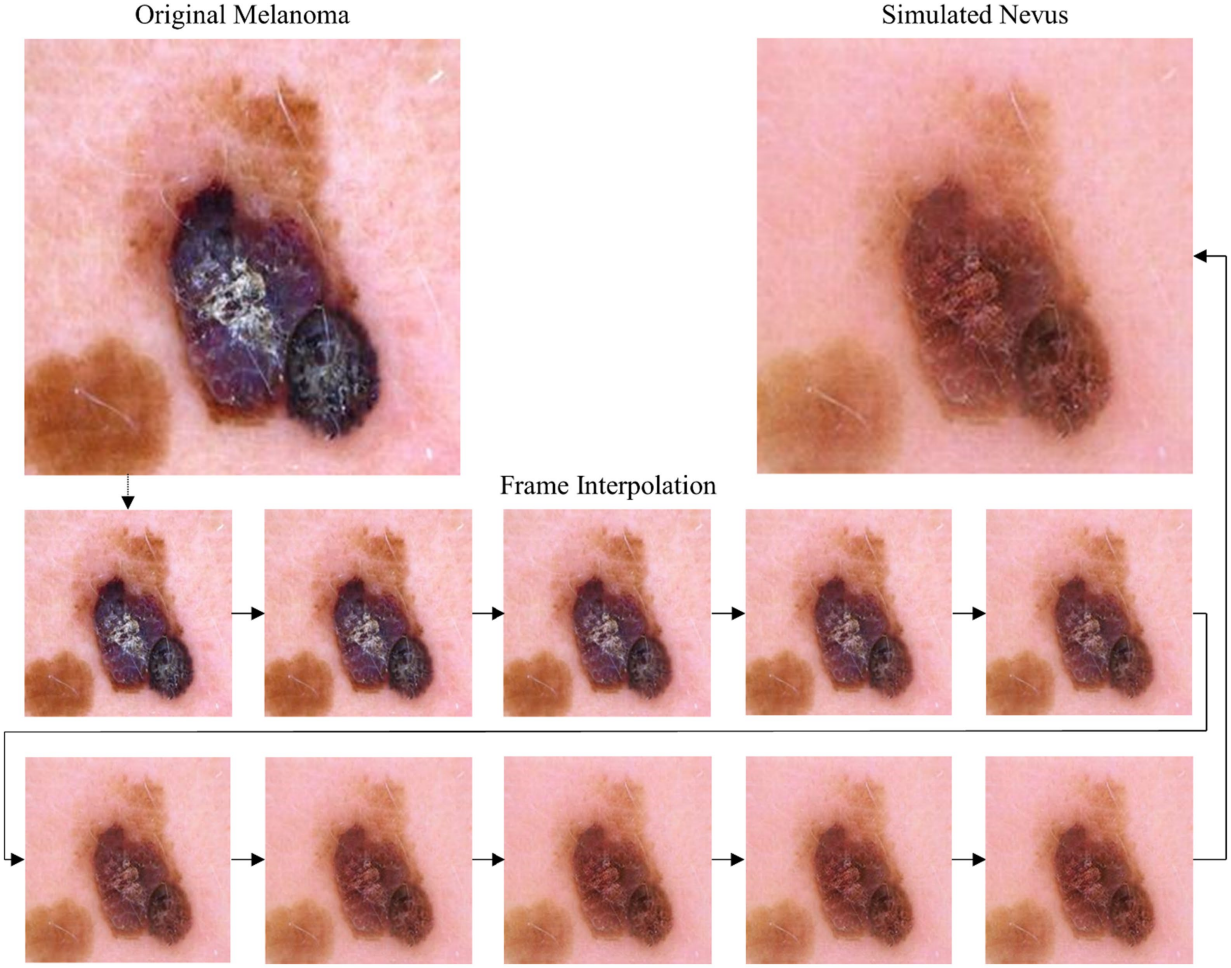
Top: Original melanoma image from ISIC dataset (left) and AI-generated image (right) showing the lesion’s potential nevus state. Bottom: Selected frames from the frame interpolation video generated with Runway (2024 Runway AI, Inc., New York, United States) showing a stepwise progression from the melanoma state to the simulated nevus state.

Conversely, Figure 4 shows an example of the backward simulation direction from a real melanoma-lesion image into a simulated nevus.

## 3 Evaluation

**Figure 5:**
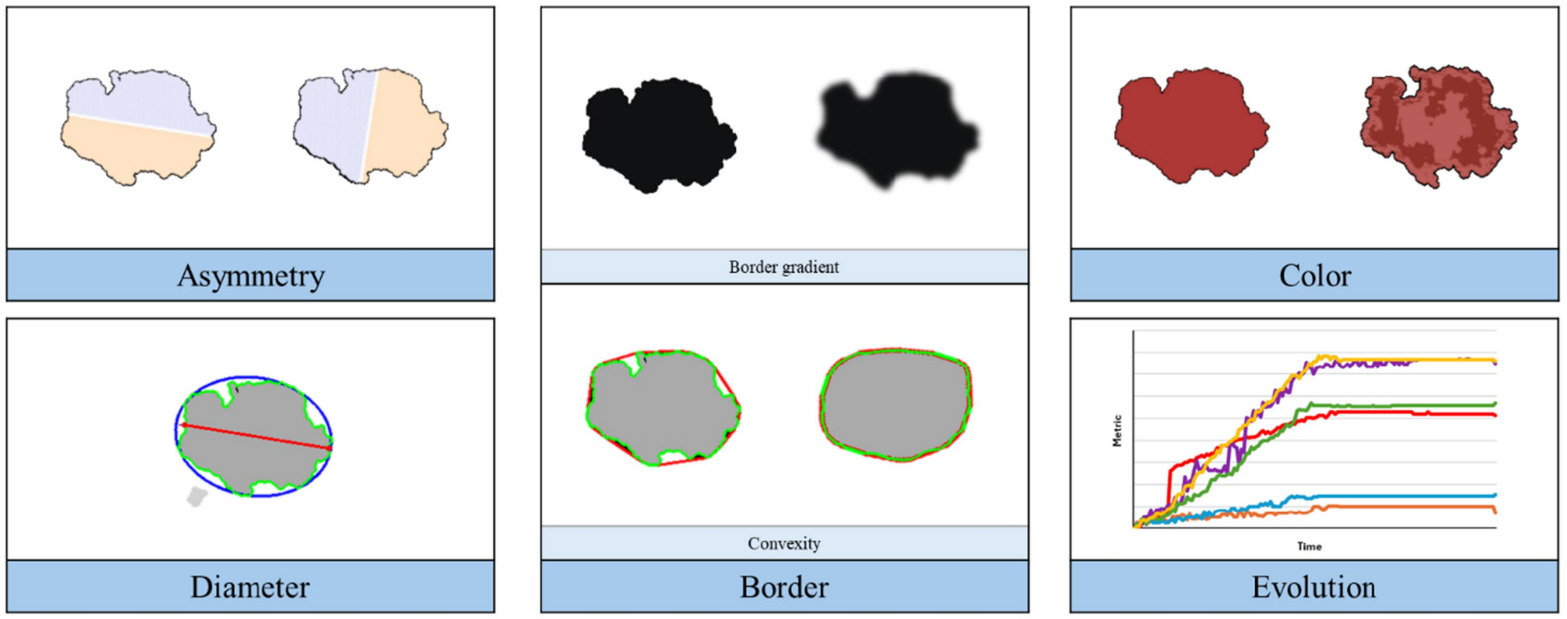
Overview of the implemented image processing techniques for ABCDE rule analysis of the simulation.

While various melanoma diagnosis scores exist, e.g., the 7-point checklist and the Menzies method (37), the ABCDE rule was utilized as example case here due to its widespread acceptance and ease of implementation via image processing. To assess how the simulated progression of skin lesions adheres to the ABCDE rule for melanoma diagnosis, we implemented the lesion properties illustrated in Figure 5 and computed them for each image in the simulation. These metrics provide quantifiable insights into various aspects of the lesion morphology, each represented by a score ranging from 0 to 1, except for the lesion diameter. The lesion diameter is presented in metric units if the image resolution (mm/pixel) is available; otherwise, it is provided in number of pixels.

In this work, we consider six different metrics, i.e., asymmetry, border gradient and convexity, normalized cluster color dispersion, diameter, and color evolution.

Asymmetry measures the degree of dissimilarity between the two halves of the lesion. This metric is calculated by dividing the lesion along its major axis, mirroring one half across that axis, and assessing their structural similarity index (38). The same process is repeated along the minor axis. The computed similarity scores are then translated into dissimilarity values, which are then averaged to derive the final metric. It is expressed on a scale where 0 signifies perfect symmetry, while 1 indicates complete asymmetry, reflecting the balance or imbalance between the lesion halves.

In our analysis, we also evaluate the border characteristics of the lesion using two metrics: border gradient and shape convexity. The gradient magnitude along the lesion contour offers insights into the sharpness or abruptness of intensity changes. This metric is computed using both the image and the segmentation mask. First, the image gradients in the x and y directions are calculated using the Sobel operator (39). These gradients are then combined to compute the gradient magnitude, which is subsequently normalized to the range [0, 1]. Next, the lesion contour is derived from the segmentation mask, and the gradient magnitude values at the image coordinates along the contour are extracted. These values are averaged to compute the final score. In our approach, low values suggest a gradual transition or smooth boundary, while high values indicate sharp transitions, emphasizing well-defined boundaries. Additionally, convexity measures the extent to which the lesion border protrudes outward, and it is computed using the following equation:


$$\mathrm{Convexity}=\frac{{\mathrm{Area}}_{\mathrm{lesion}}}{{\mathrm{Area}}_{\mathrm{convex hull}}}$$

This metric relies solely on the mole’s segmentation mask. The lesion area is determined by quantifying the number of pixels within the lesion region. Next, the convex hull of the mask is determined, representing the smallest convex shape that can completely enclose the lesion. The area of the convex hull is then computed similarly by quantifying the number of pixels within this enclosing shape. A value of 1 signifies perfect convexity, while values less than 1 denote concavity or indentations in the lesion’s shape.

We utilize the normalized cluster color dispersion as the chosen metric to quantify color properties, indicating the level of color heterogeneity or variegation within the lesion. This entails computing lesion color segmentation, for which we developed a clustering approach based on color distances in the linear RGB color space. We first test for unimodality of the lesion color distribution using Hartigan’s dip test (40). If the null hypothesis is rejected, the number of clusters is determined based on the best silhouette coefficient (41). After performing color segmentation, the Euclidean distance of each pixel’s intensity to the centroid of its assigned cluster is determined. The standard deviation of these distances for each cluster is then computed and normalized to the range [0, 1]. The final color dispersion score is derived as the weighted sum of these standard deviations, with weights corresponding to the percentage of pixels in each cluster. Values near 0 suggest a predominance of a single color, while values close to 1 indicate extreme color variation or maximal dispersion, such as black and white.

The diameter measures the length of the lesion’s longest axis. To determine this, we identify the optimal ellipse that aligns with the lesion’s shape and calculate the intersection points of its major axis line with the lesion contour. The Euclidean distance between these points represents the lesion’s diameter.

The evolution of the lesion over time can be assessed as the comparison of the previous metrics at two different time points. However, in our experiments we also evaluate the color variation by implementing a strategy to quantify changes in lesion color throughout the simulation. The lesion color segmentation explained before is firstly performed for the two captures of the lesion. Subsequently, distances between the centroids of the clusters from both segmentations are computed, based on maximum intersection over union (IoU) correspondence. These distances are then averaged and normalized by the maximum number of clusters, with values near 0 indicating subtle color changes and values near 1 representing extreme color transitions, such as from black to white.

We also employ Farneback’s method of optical flow to analyze the transformation within the simulation frames, tracking the motion between each stage of the lesion’s progression. Optical flow quantifies the apparent motion of objects, surfaces, and edges in a visual scene, based on the changes in brightness patterns between consecutive frames (42). By accumulating the optical flow vectors across all frames, we create a heatmap that visualizes the degree of change, with the magnitude of these vectors indicating the extent of transformation. Accumulated optical flow involves summing up the optical flow vectors over a sequence of frames rather than examining them pairwise. This approach can highlight consistent motion patterns across multiple frames (43).

**Figure 6:**
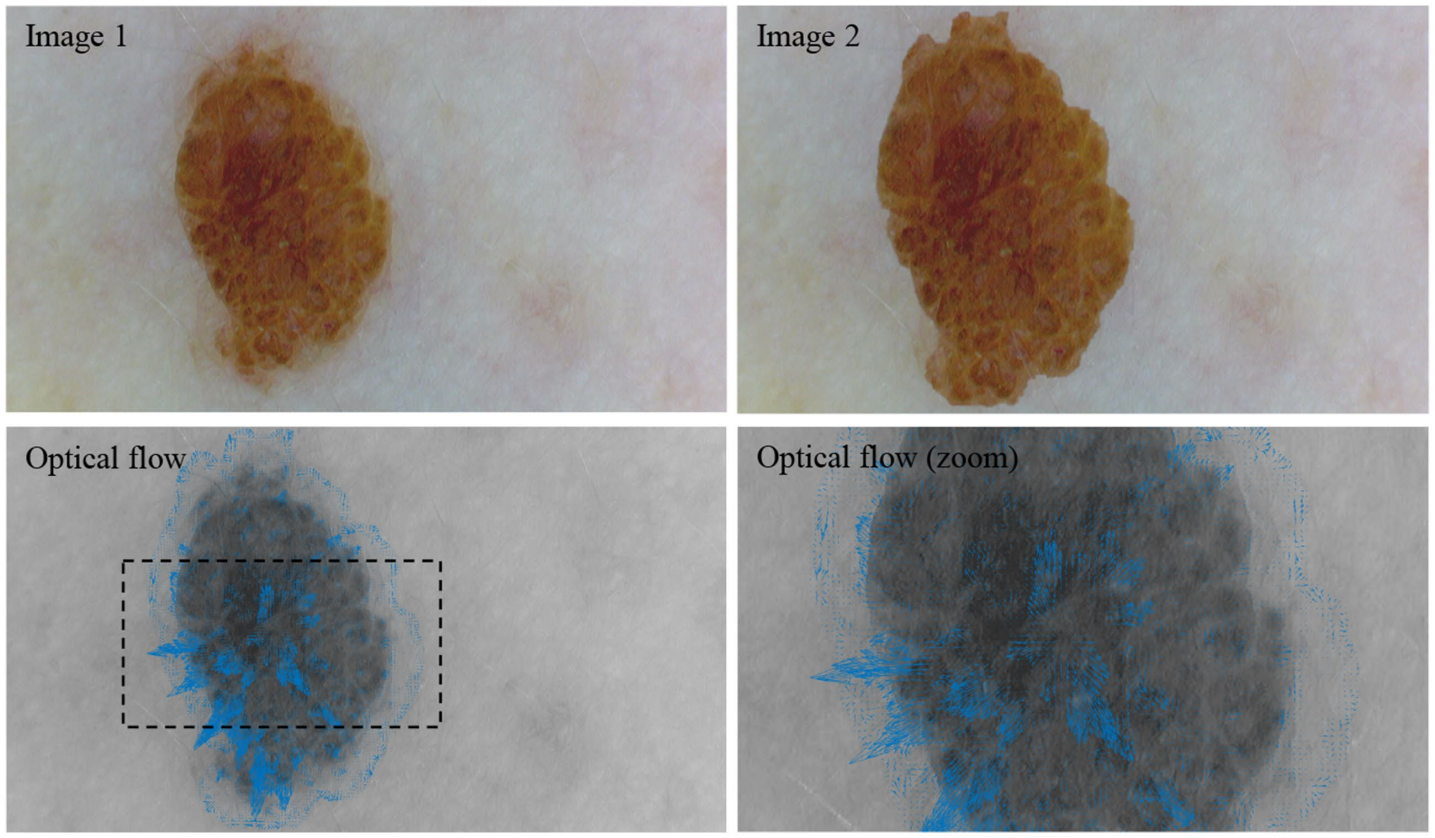
Visual representation of optical flow. Between image 1 and image 2, the lesion is cropped and enlarged. The resulting optical flow is visualized by the blue vectors.

Although this method results in the loss of the directional information between single frames, it effectively highlights regions of significant activity over the complete simulation. To complement this, we overlay the accumulated optical flow vectors on the last frame, representing the simulated melanoma state, to provide spatial context to the changes. Figure 6 shows a visual explanation of optical flow.

In Figure 6, image 1 shows a nevus acquired with a dermoscope (DE300, Firefly Global, Belmont, Massachusetts, United States). For image 2, the lesion was automatically cropped, enlarged, and superimposed onto image 1. The optical flow between image 1 and image 2 shows this enlargement as the vectors are generally pointing outwards from the center of the lesion. Please note that this example is intended solely to illustrate the concept of optical flow within a dermatological context.

To gauge the extent to which the generated images capture the likeness of nevus or melanoma lesions, we trained a classifier on real images and then analyzed its confidence on all the generated frames. This method allows us to examine the classifier’s discernment and determine the fidelity of the generated images to both nevus and melanoma characteristics.

For frame classification, we utilize a self-trained model based on the VGG11 architecture, trained on the HAM10000 dataset (44). VGG models are well known for their effectiveness in transfer learning and have been previously used in skin cancer classification models (45). The dataset is labelled with seven different skin lesion types (benign classes: melanocytic nevi, benign keratosis-like lesions, dermatofibroma, vascular lesions; malignant or pre-malignant classes: melanoma, basal cell carcinoma, actinic keratosis). The model was trained for 15 epochs, to distinguish the skin lesions from the melanocytic nevi class (5,759 images) and the melanoma class (956 images). We evaluate the model performance on a validation dataset randomly selected from the HAM10000 dataset. This consists of 1,103 images (melanocytic nevi: 946, melanoma: 157); the validation data has not been used to train the classification model. The accuracy score of 0.9 is derived from the total number of images that were correctly identified as nevus or melanoma.

The samples chosen for this analysis were carefully selected from the dataset to ensure a broad representation of diversity and variability in lesion types. This selection was based on criteria such as relative lesion size, lesion color variation, and skin color to provide a comprehensive evaluation of our methodology across different scenarios.

## 4 Results

**Figure 7:**
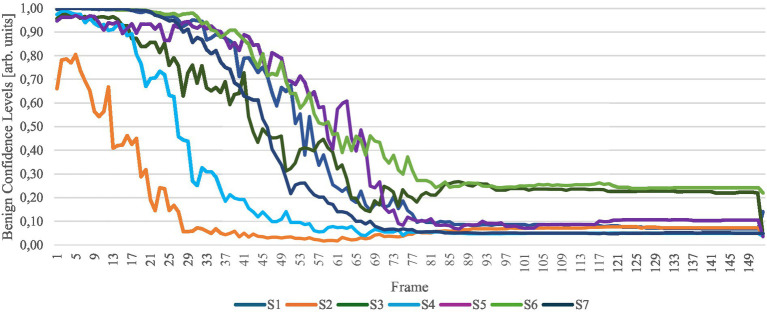
Confidence levels of a nevus diagnosis for each frame. The *y*-axis quantifies the model’s confidence in each diagnosis, reflecting the degree of certainty associated with the classification outcomes. S1 to S7 represent the samples in the study.

The classification model’s confidence in diagnosing each frame is shown in Figure 7. The confidence value reflects the model’s certainty in its classification decision.

The confidence levels indicate that the classification model initially accurately identifies the nevus input image as a nevus lesion. As the simulation progresses, the model’s confidence in this diagnosis decreases. Conversely, as the combined confidence levels always approximate a total of 1, the confidence for melanoma increases. This shift effectively highlights the model’s capability to simulate the potential evolution of a lesion from nevus to melanoma.

Beyond a certain number of frames, the classification remains largely unchanged, likely because the frame interpolation no longer significantly alters the image, suggesting that only subtle modifications are made beyond this point. It is important to note the significant confidence difference observed between the penultimate frame and the last frame. This variation arises because the first and last frames, unlike the interpolated frames, are not influenced by frame interpolation effects. The interpolation process introduces a level of noise absent in the training data used for the classification model, which could explain the discrepancy in model confidence. However, the transition in confidence from the first frame to the second frame—the initial frame produced by interpolation—is not as pronounced, indicating a gradual adaptation of the model to the interpolated data.

**Figure 8:**
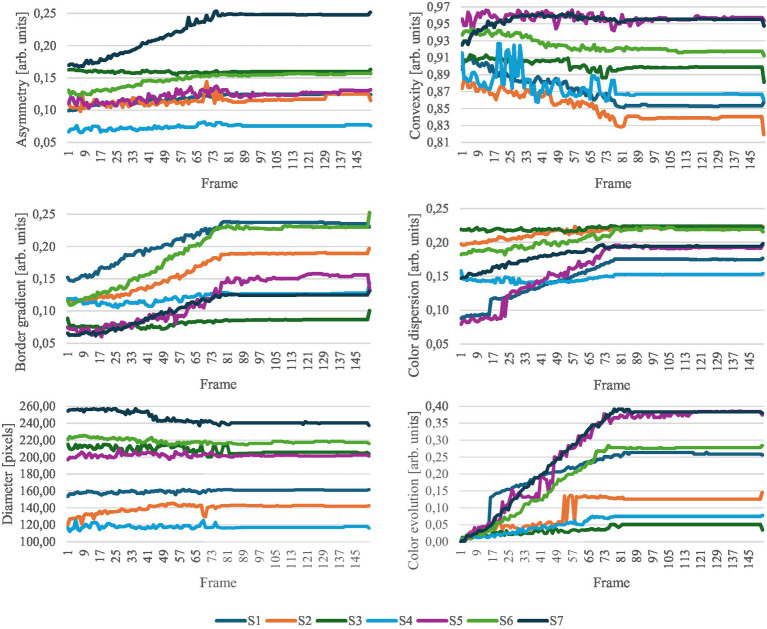
Evolution of ABCDE rule metrics. S1 to S7 represent the samples in the study.

In the following, we aim to determine how the simulation of melanoma progression conforms to the widely recognized ABCDE rule for melanoma diagnosis. Figure 8 displays the evolution of the lesion evaluation metrics for each frame throughout the simulation.

Generally, the latter half of the simulation exhibits minimal changes, which was also observed in the confidence values. The results appear relatively noisy, highlighting the strong dependence of the metrics to the segmentation masks. This issue could be mitigated by improving the generation of segmentation masks for the simulation. Currently, each frame is segmented independently, without considering the continuity with previous frames. Introducing a method that ensures segmentation masks maintain consistency across successive frames could significantly reduce noise and enhance data quality.

Within each metric category, certain samples are more significantly impacted by changes related to that specific metric. For example, the asymmetry increases significantly more for sample S7 than for the other samples. The border gradient increases throughout the simulation, suggesting more defined borders in the simulated melanoma state. However, this is contrary to the expectations set by the ABCDE rule, which typically associates melanoma with less defined boundaries. Regarding color metrics, there is a notable emphasis on color changes, evolution, and dispersion, indicating that the simulation predominantly modifies color aspects of the lesions. A strong increase in the lesion diameter is observed in S2. For the other samples, the diameter undergoes only a small change. The analysis of these metrics highlights the complex interplay among parameters for the early detection of melanoma. In clinical dermatology, not all parameters of the ABCDE rule need to be met to justify an excision and melanoma lesions do not necessarily exhibit all ABCDE characteristics simultaneously. Similarly, in our simulations, adherence to the metric parameters varies across samples; some samples may show a pronounced change in one metric while others remain unchanged. This variability mirrors the clinical reality, underscoring the simulation’s relevance and applicative value in dermatological assessments.

**Figure 9:**
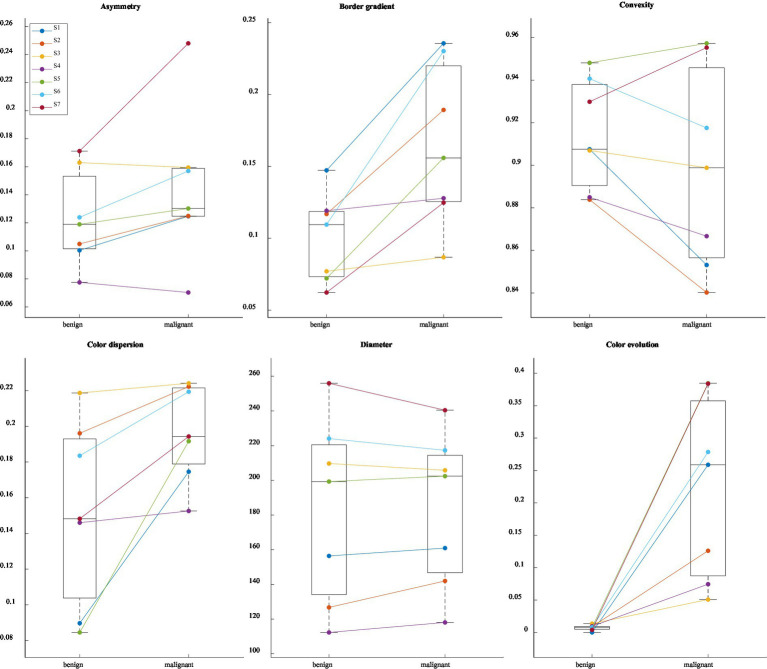
Paired boxplot for each ABCDE rule related metric with the first and last frame of the frame interpolation. S1 to S7 represent the samples in the study.

Figure 9 shows statistical trends for the first and the last frame of the frame interpolation.

The paired boxplot for the first and the last frame of the simulation reveals general trends across the dataset, while also highlighting variations among individual metrics. Particularly in color-related metrics and the border gradient, a consistent behavior is observed across all samples. However, the plot also underscores the absence of absolute metric values defining nevus and melanoma states; metrics indicative of a nevus in one sample may correspond to a melanoma in another, illustrating the complex and variable nature of these diagnostic indicators.

The Pearson correlation coefficients (PCCs) and *p*-values presented in Table 1 demonstrate the degree of correlation between the evolution of each sample and the respective classification confidence, as illustrated in Figure 7.

**Table 1:** Pearson correlation coefficients and *p*-values.

	S	A	B		C	D	E
		Asymmetry	Gradient	Convexity	Color	Diameter	Evolution
*ρ*	**1**	−0.954702	−0.968174	0.954087	−0.961917	−0.800910	−0.875324
*p*-val		0.000000	0.000000	0.000000	0.000000	0.000000	0.000000
*ρ*	**2**	−0.450438	−0.60801	0.601743	−0.782247	−0.846785	−0.643279
*p*-val		0.000579	0.000000	0.000000	0.000000	0.000000	0.000000
*ρ*	**3**	0.297445	−0.781473	0.822825	−0.727357	0.601767	−0.849163
*p*-val		0.000198	0.000000	0.000000	0.000000	0.000000	0.000000
*ρ*	**4**	−0.674758	−0.652609	0.590950	−0.447553	0.181571	−0.905309
*p*-val		0.000000	0.000000	0.000000	0.000743	0.025174	0.000000
*ρ*	**5**	−0.752571	−0.961138	0.306781	−0.930462	0.113930	−0.954466
*p*-val		0.000000	0.000000	0.000121	0.000000	**0.162240**	0.000000
*ρ*	**6**	−0.945634	−0.989335	0.931796	−0.970484	0.760012	−0.980060
*p*-val		0.000000	0.000000	0.000000	0.000000	0.000000	0.000000
*ρ*	**7**	−0.983136	−0.983924	−0.427019	−0.956945	0.977155	−0.982438
*p*-val		0.000000	0.000000	0.000411	0.000000	0.000000	0.000000

The font color indicates the sign of the correlation (red: negative, black: positive). A gray field shows that the sample’s correlation has a different sign in that category than the expected according to the ABCDE rule. A bold field indicates a high *p*-value. S1 to S7 represent the samples in the study.

**Figure 10:**
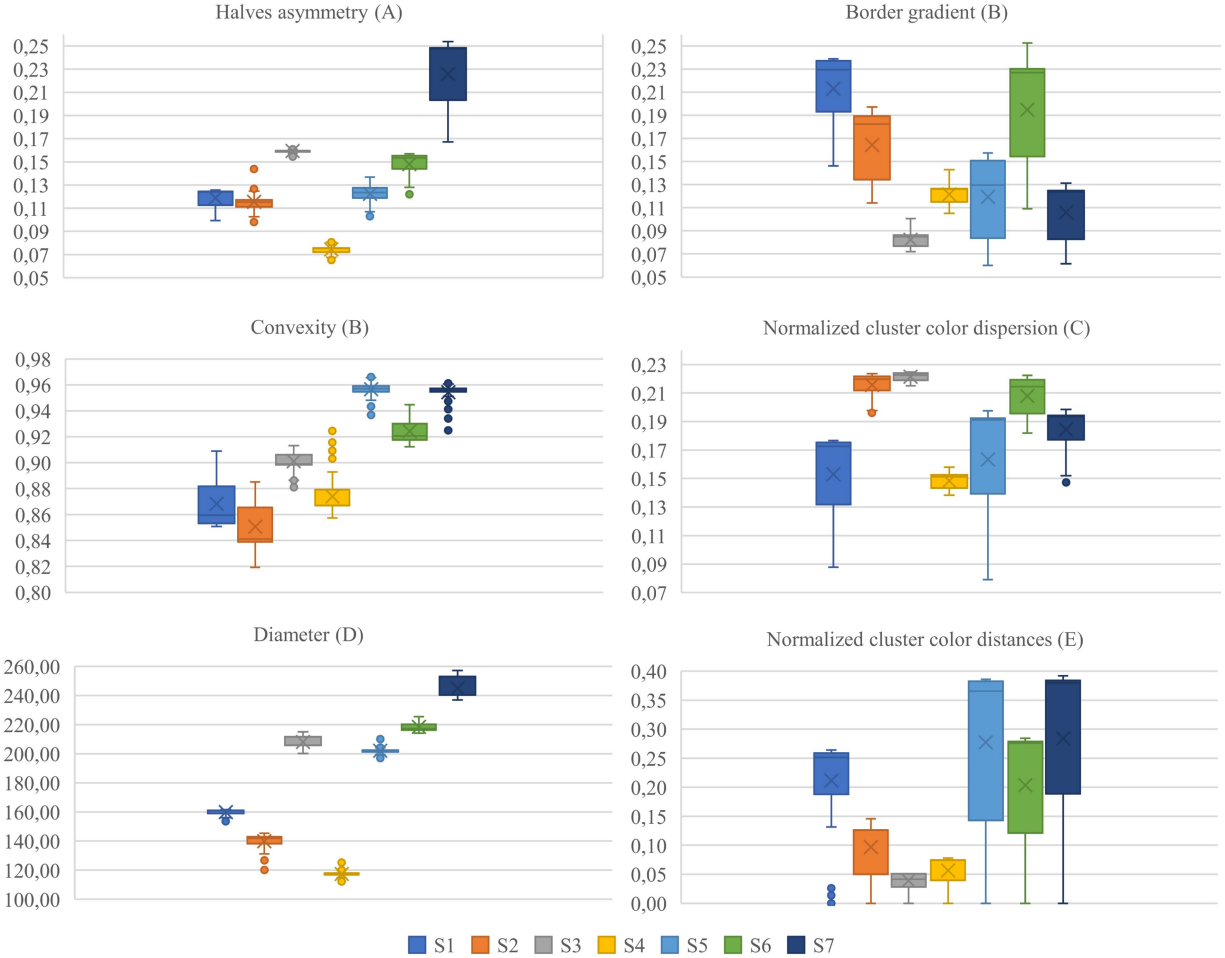
Value distributions for each metric and sample for full simulations. S1 to S7 represent the samples in the study.

Table 1 reveals numerous significant correlations across all metrics, with the *p*-value exceeding the threshold of 0.05 only once. Asymmetry, border characteristics, color dispersion, and color evolution exhibit strong correlations. In contrast, the correlations for convexity and diameter are less pronounced. The boxplots displayed in Figure 10, which illustrate the value distribution of metrics for the complete simulations, aid in understanding why certain samples and metrics correlate with the classification confidence while others do not.

Focusing on the samples and metrics that exhibit correlations in the opposite direction of those anticipated by the ABCDE rule (highlighted in gray in Table 1), we observe that the dispersion of the metric distribution for these cases is small, indicating almost imperceptible changes for asymmetry, convexity, and diameter, as illustrated in Figure 10. These subtle variations could be attributed to the inherent variability of the segmentation masks, suggesting that the detected changes are not significant.

On the other hand, for the border gradient metric, we observe strong negative correlations, indicating that the lesion borders become sharper as the simulation progresses, contrary to what is expected by the rule. This could be attributed to the characteristics of the training data or the model’s focus on certain features. Observing the original and simulated images depicted in Figures 2, 3, we notice that the lesions tend to become darker and sharper as the simulation progresses, which aligns with the results obtained.

For the rest of the samples and metrics, we see that color-related parameters, such as normalized cluster color dispersion and normalized cluster color distances, along with border gradient, exhibit a broader distribution compared to other parameters like diameter. The wide dispersion of these metrics indicates that the simulations have a pronounced impact on color.

**Figure 11:**
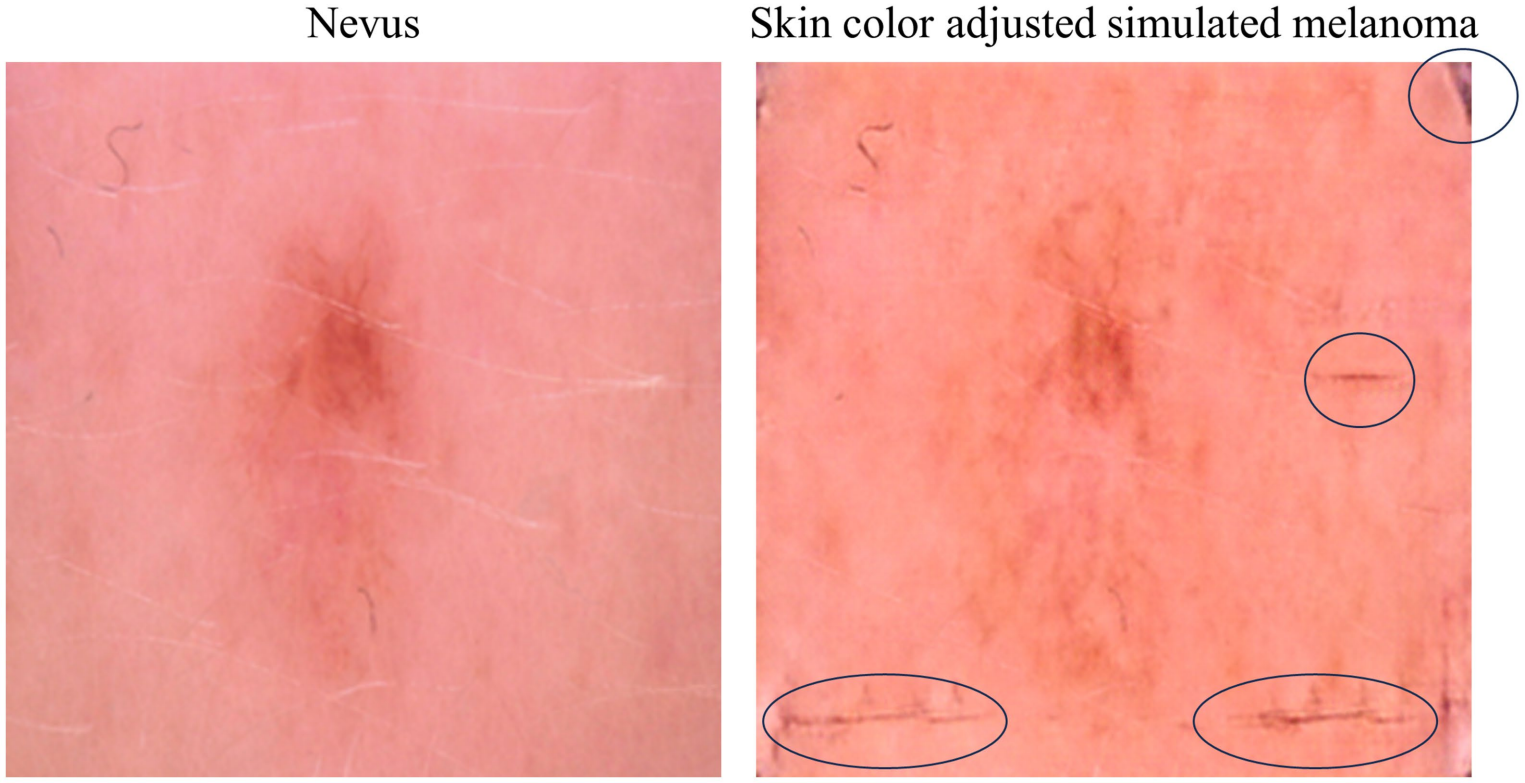
Original nevus image (left) and simulated melanoma image (right), highlighting a case where artifacts and border effects are present in the simulation.

In rare cases, the simulation produced undesired artifacts and border effects, which can reveal the computational nature of the generated image. Figure 11 illustrates an example where such artifacts and border inconsistencies are present.

These artifacts and border inconsistencies highlight a limitation of the current simulation model, which will need to be addressed in future work by developing post-processing filtering methods that can quantify the degree of artifact interference and incorporating this feedback into the model training, ensuring that future iterations produce fewer such artifacts.

**Figure 12:**
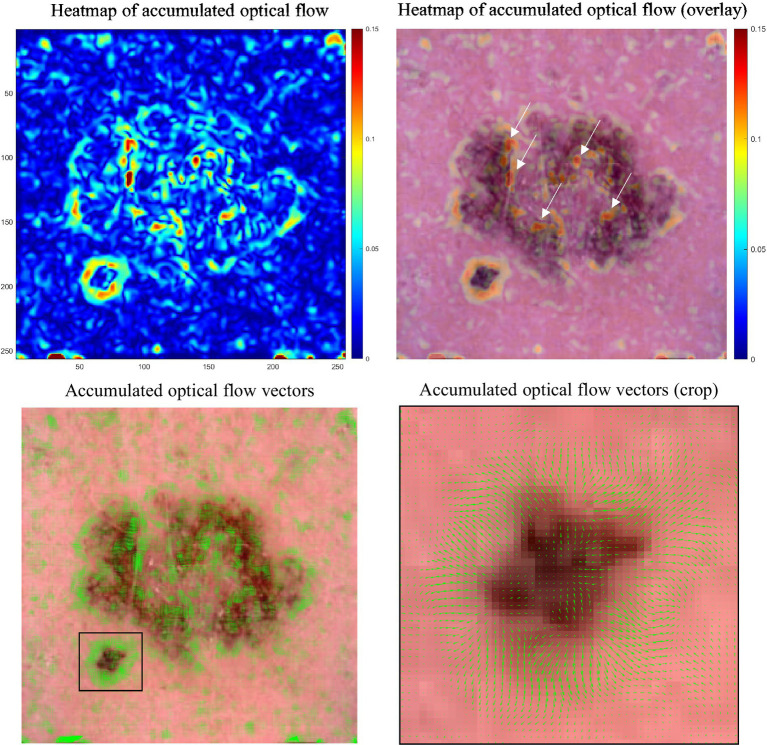
Optical flow analysis of lesion evolution. Upper left: Heatmap of accumulated optical flow vectors, with color intensity indicating change magnitude and highlighting transformation areas. Upper right: Heatmap overlaid on the simulation’s final, simulated melanoma frame, combining flow data with visual context. Lower left: Optical flow vectors across the lesion, emphasizing motion. Lower right: Zoomed-in view of vectors from the lower left, revealing detailed motion patterns within a focused area.

Figure 12 illustrates the dynamics of lesion evolution, employing optical flow analysis to map the transition from a nevus to a simulated melanoma.

The heatmap of the accumulated optical flow visualizes the degree of change a lesion region undergoes in the simulation. The degree of change varies across different parts of the lesion, with some areas showing significant transformation while others exhibit minimal changes. This can be seen in the overlay of the heatmap and the last frame of the simulation (Figure 12, upper right) as the red color signifies a strong degree of change only for certain regions in the lesion (see white arrows) while other regions undergo less change which is visualized by the green and blue colors in the heatmap.

The accumulation of optical flow vectors provides insights into the subtle movements and overall expansion of the lesion. Although the vectors are small, their accumulation across the lesion reveals patterns of growth and transformation. The zoomed-in view highlights the detailed motion patterns, showing the outward orientation of vectors that indicates expansion in specific parts of the lesion.

While this information does not directly impact the diagnosis, it could be valuable monitoring lesion progression. Consequently, the resulting vector field from optical flow analysis opens opportunities for advanced vector field analysis. This includes exploring fixed points (sink, source, saddle), periodic orbits (attracting, repelling), and vector field topology which could improve the understanding of lesion progression dynamics.

## 5 Discussion

Comparing our findings with existing literature, such as Stanganelli et al. (46), we observe that digital monitoring has proven pivotal in the early detection and management of melanocytic lesions. Stanganelli et al. highlighted the critical role of digital monitoring in identifying subtle changes in atypical lesions, which aligns with our approach of simulating lesion evolution to visualize these changes dynamically. Their results showed that frequent digital monitoring significantly aids in early melanoma detection and reduces unnecessary excisions, especially in high-risk patients.

Similarly, Kittler et al. (47) demonstrated the effectiveness of sequential dermoscopy imaging (SDI) in detecting early melanomas that lack specific features at baseline. Our method offers a dynamic perspective on lesion evolution, complementing traditional SDI techniques. Altamura et al. (48) found that short-term SDI at three-month intervals effectively identifies melanomas lacking dermoscopic features. After 6 weeks, already 69% of the melanomas were detected. However, 31% of melanomas required the full monitoring period for detection, highlighting the need for prolonged observation for some lesions.

Haenssle et al. (49) emphasized the importance of individualized surveillance plans based on melanoma risk factors, showing that high-risk patients benefit significantly from frequent digital dermoscopy follow-ups, leading to the detection of thinner melanomas at earlier stages. Similarly, Argenziano et al. (22) noted that dermoscopic monitoring increases the likelihood of detecting featureless melanomas and minimizes unnecessary excisions of benign lesions. Their study also found that short-term monitoring protocols at three-month intervals had the highest patient compliance and were effective in detecting early melanomas, although slow growing melanomas required longer monitoring periods to reveal changes.

Terushkin et al. (50) on the other side found that slow-growing melanomas often exhibit minimal growth over long-term follow-up, becoming more disorganized and developing new colors and structures over time. This aligns with our findings that prolonged monitoring is crucial for detecting subtle changes indicative of melanoma. Salerni et al. (51) analyzed the benefits of a two-step method (total-body photography and digital dermatoscopy) for early melanoma diagnosis, emphasizing the need for high patient compliance and resource-intensive follow-up programs. In our study we address these challenges by providing a dynamic and detailed perspective on lesion evolution.

Also, Argenziano et al. (21) stressed the importance of understanding the natural evolution of melanocytic lesions and improving melanoma diagnosis through dermoscopy and digital follow-up. Our approach addresses this issue by potentially providing a more detailed and dynamic visualization of lesion changes over time.

Overall, our study aligns with and extends the existing literature by demonstrating the effectiveness of a dynamic monitoring approach in improving the early detection of melanoma and reducing unnecessary surgical procedures. This method provides a comprehensive understanding of lesion evolution, addressing key challenges identified in previous studies and offering a promising tool for enhancing melanoma surveillance and diagnosis. Further systematic studies will have to follow, however, to demonstrate the full potential of the approach.

Buhl et al. (52) discusses an approach to improve melanoma detection by combining static and dynamic dermatoscopic features. The DynaMel algorithm was developed through a prospective observational study involving 688 patients at high risk for melanoma, with a follow-up period averaging 44.28 months. During this time, 675 lesions exhibiting dynamic changes were excised, leading to the identification of 61 melanomas. The study found that integrating dynamic criteria - such as asymmetric multifocal enlargement, focal changes in pigmentation, and overall pigmentation changes - into the traditional 7-point checklist significantly increased the sensitivity of melanoma detection from 47.5 to 77.1%, while maintaining a high specificity. Our study ties in well with this work as it simulates the dynamic changes in lesions.

## 6 Conclusion

This research explores the potential of combining lesion progression simulations with optical flow analysis to provide dermatologically relevant insights on the lesions examined. By adhering closely to the ABCDE rule, the simulations prove to be robust tools for representing the evolution of skin lesions, providing dermatologists with a reliable method to visualize and understand disease progression. Optical flow analysis further adds to this approach by highlighting dynamic changes within the lesions, identifying areas that require higher clinical attention during lesion monitoring. Beyond simply visualizing the changes, optical flow offers a quantitative assessment of lesion movement and expansion patterns, which may be particularly useful in detecting early-stage melanomas where traditional visual cues may be insufficient. This approach not only complements the ABCDE rule but could also evolve into a new diagnostic layer that quantifies lesion transformations in real-time, potentially serving as an early warning system for rapid melanoma progression. Integrating such quantitative tools into routine dermatological practice may lead to earlier interventions, personalized follow-up schedules, and more precise treatment plans for patients at risk of melanoma. However, the potential of optical flow analysis will need to be studied in future work in more detail.

This visual tool goes beyond the traditional ABCDE rule for melanoma diagnosis, offering a more intuitive and understandable approach. By seeing the potential progression from nevus to melanoma, patients gain a clearer understanding of their diagnosis, fostering confidence in self-examinations and early detection. Melanoma patient education on skin self-examination improves their self-efficacy. With this, the level of perceived physician support increases (53). Generative AI in dermatology is not just a technological advancement; it could be a step towards empowering patients with a deeper understanding of their skin health. By bridging the gap between complex melanoma diagnostics and patient comprehension, technology could enhance proactive skin care and early cancer detection. The broader application of this technology could improve patient education across various diseases, that require visual diagnosis. Deploying AI in patient care and education necessitates careful consideration of ethical issues, including patient privacy, data security, and the need for transparent AI decision-making processes (54).

Furthermore, while the simulation operates independently of the ABCDE rule, its outcomes correlate with this established diagnostic framework, underscoring the potential of generative AI to mimic complex biological processes. This allows for testing and validating novel diagnostic criteria against the simulations. Such an approach could not only enhance our understanding of skin lesion progression, but also lays the groundwork for incorporating new, evidence-based criteria into clinical practice, potentially improving the early detection of melanoma. When considering the use of this technology as a training tool for dermatologists and an educational tool for patients, it is important that the simulation impacts the parameters in a natural and realistic manner. This ensures that the learning experience is authentic and reflective of real-world conditions, which is precisely what our simulation achieves. Finally, the simulation could be helpful in developing algorithms specifically designed for lesion change detection, enhancing the early diagnosis of melanoma and monitoring of skin lesion progression.

While it is recognized that the majority of melanomas do not originate from benign melanocytic nevi, this approach is specifically tailored to enhance detection and monitoring of those melanomas that develop from pigmented skin lesions (55).

The simulations effectively adhere to the ABCDE rule, affirming their validity in mirroring realistic lesion dynamics. The optical flow analysis promises applicability in identifying regions within the lesions that require heightened attention by the dermatologist.

Future challenges include enhancing the resolution of the input and output images (presently at 256 × 256), which is crucial for improving detail and clarity. Additionally, more research is needed to minimize artifacts and border effects that may distort results.

In future work, we plan to validate the generated lesions by acquiring a cohort of board-certified dermatologists to independently diagnose the images, thereby ensuring the clinical relevance and accuracy of our simulations. We plan to explore the generalizability of the approach by applying the Cycle-GAN to other dermatological conditions (e.g., basal cell carcinoma) and other medical imaging datasets (e.g., microscopy or histopathology) to evaluate its performance and potential across different contexts.

Our next work will prioritize comparing these simulations with actual lesion progressions from sequential dermoscopy, which is challenging due to the rarity of such data, as suspicious lesions are often excised preemptively. The technology could be implemented into patient consultations, explaining the necessity of excisions, and assessing patient confidence in dermatological decisions.

## Data Availability

The raw data supporting the conclusions of this article will be made available by the authors, without undue reservation.
